# Narcissism but Not Criminality Is Associated With Aggression in Women: A Study Among Female Prisoners and Women Without a Criminal Record

**DOI:** 10.3389/fpsyt.2019.00021

**Published:** 2019-02-01

**Authors:** Georgia Kalemi, Ioannis Michopoulos, Vasiliki Efstathiou, Foteini Konstantopoulou, Domna Tsaklakidou, Rossetos Gournellis, Athanassios Douzenis

**Affiliations:** Forensic Unit, 2nd Department of Psychiatry, Medical School, National and Kapodistrian University of Athens, University General Hospital Attikon, Athens, Greece

**Keywords:** narcissism, self-esteem, aggression, self-perception, criminality, female inmates, prison, type of offense

## Abstract

Aggression has drawn research attention during the past decades. It remains unclear how self-esteem, self-perception, narcissism and certain socio-demographic factors impact the course of aggression. Female aggression is considered to differ in its origins and is understudied. Only few studies have attempted to examine the aforementioned variables among females, while none of them included a comparison between delinquent and non-delinquent individuals. The present study examines the effect of self-esteem, self-perception, narcissism, and socio-demographic factors on aggression among female inmates and women without criminal record (non-delinquents). One hundred fifty-seven female inmates in the Attica's Korydallos Female Prison and 150 women with no criminal record were assessed with Buss & Perry Aggression Questionnaire, Rosenberg's Self-esteem Scale, Narcissistic Personality Inventory-40 and the Self-Perception Profile for Adults. When inmates were compared to non-delinquent women, it emerged that higher aggression could be independently predicted by higher levels of narcissistic personality traits and sociability, as well as lower age, lower education, lower self-esteem, and lower levels of self-perception items including nurturance, job competence and athletic abilities. Aggression was not predicted by the participants' group (inmates vs. non-delinquents). Within female inmates, independently of the type of their offense (convicted for violent vs. non-violent crimes), it was found that lower job competence, higher narcissistic personality traits and a history of childhood maltreatment could predict higher aggression. Our results support the notion that female aggression differs from male and highlight the significant parameters that may predict aggression either among women (inmates and non-delinquent women) or among female inmates (violent or non-violent crimes). It is the presence of narcissistic traits which predict aggression rather than criminality in general, including violent and non-violent crimes.

## Introduction

Aggression is defined as any behavior toward another individual with clear intent to cause harm (1). It is quite challenging to establish a solid theoretical and practical framework for aggression and therefore research studies differ significantly in terms of their conceptualization and methodology (2). Women seem to express aggressive behavior differently than men (3, 4).

In a meta-analytic review of sex differences in aggression they found that although men were more aggressive than women on the average, sex differences were inconsistent across studies. The tendency for men to aggress more than women was more pronounced for aggression that produces pain or physical injury than for aggression that produces psychological or social harm. In addition there are sex differences with respect to the cognitive attributions related to aggressive acts. More specifically women, more than men, perceive that enacting a behavior would produce harm to the target, guilt, and anxiety in oneself, as well as danger to oneself. The results emphasize that aggression sex differences are a function of perceived consequences of aggression that are learned as aspects of gender roles and other social roles (5). Women mainly use indirect, relational aggression, and when expressing physical aggression they are more likely to have antisocial personality disorder with possible comorbid depression, anxiety or feelings of loneliness. One form of aggressive expression, which has received increased theoretical and research attention, is criminal behavior; nevertheless, female criminality is generally a neglected field of research (6–8). The number of prisoners worldwide is estimated to be over 10.2 million and although women are traditionally under-represented, there seems to be an increasing trend of women in custody and detained women (9, 10). According to the 3rd edition of World Female Imprisonment List, the number of detained women has increased by 50% compared to 2000 with this growth not corresponding to an increase of the total prison population globally (11). In Greece, according to the statistics presented by the Hellenic Police and the Ministry of Justice, from the total prison population the rate of female criminality was 6.5% in 1973, while in 2012–2015 it increased reaching 11%. Furthermore, the most frequently committed types of crimes by women, during the 4-years time period 2012–2015 were theft and robbery (*n* = 19,902), violation of the Law on Drugs (*n* = 9,716), and rarely homicide (*n* = 240) (12).

Among the socio-environmental risk factors identified to contribute to the expression of female aggression are unemployment, low socioeconomic status, poverty and the lack of access in educational and professional opportunities (13). A substantial amount of research indicates that violent women offenders have been, at some point in their life, victims of violence themselves. Greenfeld and Snell (14) found that among female offenders convicted for violent crimes, 60% reported physical or sexual abuse during childhood, while 35% during their adulthood, as victims of inter-partner violence (14). A common belief is that aggressive behavior is a personality characteristic related to self-esteem, self-perception and narcissism.

Due to the lack of relevant studies with female samples it is being assumed that women conform to similar patterns of aggression, violence, and other psychological constructs such as self-esteem, self-perception and narcissism which affect the pathway to aggression. The concept of “self” and its core values have drawn increased research attention during the past few decades. Self-perception has been defined as a cognitive aspect of the “self” which is interrelated with self-image and self-esteem and is generally referred to the “whole” of a complex, dynamic and structured system which entails beliefs, attitudes and behaviors representative of an individual's personality (15). Although there are limited research findings regarding self-perception of female offenders, it is evident that female criminality is related to gender stereotypes and women's self-perception (16–19). Self-esteem, one of self-concept's key components, is crucial for the formation and development of personality (20–23).

Findings concerning the possible connection between self-esteem and aggression are inconsistent and the nature of the relationship remains unclear. The majority of studies suggest that low self-esteem is connected to higher aggression (24, 25). Nevertheless, a number of studies have also found an association between high self-esteem and aggression (25, 26). There are also studies that reveal no association between self-esteem and aggression. Especially, in a comparison of violent and non-violent offenders, Beesley and McGuire found no differences in the levels of self-esteem (27).

Narcissism can be conceptualized as either a dimensional or a categorical trait (Narcissistic Personality Disorder in the DSM-5, American Psychiatric Association, 2013). It is worth mentioning that the present study views narcissism as a personality trait. Narcissism is associated with feelings of elevated self-worth and superiority. Narcissistic individuals tend to be overwhelmed when they perceive threat toward their self-image and they carry out maladaptive strategies in response to this distress (28–31). Violent men tend to have an increased sense of superiority and therefore are more sensitive toward factors which seem to undermine their pride and self-image. Narcissism is considered a major cause of aggression and as evident in a study among male prisoners, the scores of narcissism where higher among inmates compared to non-inmates, whereas the self-esteem scores were comparable to scores from the general population (32).

Among the most prominent theoretical models which explain the relationship between narcissism and aggression are the psychodynamic mask model (33, 34) and the threatened egotism model (32). According to the psychodynamic mask model, individuals hide their low self-esteem behind an elevated sense of self (35). The threatened egotism model suggests that it is not the presence of low self-esteem which is directly linked to aggression rather than the presence of a threat to one's self-esteem which can result in aggression (32). Although both models agree that individuals with narcissistic traits tend to have a fragile self-esteem, they differ on the impact that self-esteem has on the relationship between narcissism and aggression. On the other hand, Donnellan et al. proposed that narcissism alone does not affect the relationship between self-esteem and aggression (24).

Literature has yielded inconclusive and contradictory results with respect to the impact of self-perception, self-esteem, and narcissism on aggression and their interrelation. To the best of our knowledge, only a few studies attempted to examine those variables among female inmates, while none of them included a comparison between delinquent and non-delinquent individuals (36). Therefore, we aim to explore the effect of self-esteem, self-perception, narcissism, and socio-demographic factors on aggression among female inmates (violent or non-violent crimes) and women without criminal record.

## Materials and Methods

### Participants

In the present study a total of 307 women participated: 157 (51.1%) inmates in the Female Prison of Korydallos, situated in the prefecture of Attica, Greece and 150 (48.9%) women without a criminal record (non-delinquents).

The study was approved by the Ministry of Justice, Transparency and Human Rights and the Ethics Committee of “Attikon” University Hospital and was carried out in accordance with the Declaration of Helsinki.

Main exclusion criteria were inability to comprehend the Greek language, having a psychiatric history of psychosis, receiving psychiatric medication before arrest or at the time of interview (including illegal substances and alcohol). Women with personality disorders and history of receiving psychiatric medication 1 year before arrest were included. Women without a criminal record were randomly selected from the general population of Attica, Greece after implementing the aforementioned exclusion criteria.

Participants were recruited during a 2-years period. They all completed the Buss and Perry Aggression Questionnaire (BPAQ), Rosenberg's Self-Esteem Scale (RSES), Narcissistic Personality Inventory-40 (NPI-40), and the Self-Perception Profile for Adults.

The participants were interviewed after giving their consent and demographic and social characteristics were collected. On the following day the questionnaires were individually administered. The participants were informed that all information obtained in this study would be kept strictly confidential and anonymous, and signed an informed consent form. The participation of all the individuals was voluntary, without any financial compensation.

Overall 182 inmates were asked to participate. Two were suffering from psychosis at the time and four from major depression and were excluded. Furthermore, 12 inmates were not included due to insufficient knowledge of Greek language which did not allow them to fully understand the questions and 7 inmates did not sign the consent form. From the control sample, 168 women were asked to participate. Eleven refused to participate and 7 were excluded due to insufficient knowledge of Greek language.

### Assessment Instruments

#### Rosenberg's Self-Esteem Scale (RSES)

The RSES is a 10-item self-report questionnaire, which evaluates feelings of self-worth and self-esteem, with items answered on a four-point scale, from “strongly agree” to “strongly disagree.” Scores range from 0 to 30, with higher scores indicating higher self-esteem (37). RSES has been translated and adapted in Greek and has been widely used (38). In the current study, RSES showed very good internal consistency (Cronbach's α = 0.866).

#### Buss and Perry Aggression Questionnaire (BPAQ)

The BPAQ is a 29-item self-report questionnaire which has been designed to measure aggression as a trait. It is answered on a 5-point scale and the scores range from 29 to 145, with higher scores suggesting higher aggression. BPAQ has been translated and validated in Greek (39). In this study, BPAQ showed excellent internal consistency (Cronbach's α = 0.905).

#### The Narcissistic Personality Inventory-40 (NPI-40)

The NPI-40 is a 40-item self-report measure that conceptualizes total scores for narcissism across seven components (40). The NPI-40 is a multiple-choice response questionnaire, where the participants choose between two options which best describes them. Scores range from 0 to 40, with higher scores indicating higher narcissism. Two psychologists, bilingual in Greek and English translated NPI-40 from the English version into Greek. They examined the two drafts and chose the expressions that best conveyed the sense of the original. The new Greek text agreed, was given to a bilingual Greek psychiatrist who translated it back into English. The two versions were found to be identical in terms of content, with minor grammatical differences. In this study, NPI-40 showed very good internal consistency (Cronbach's α = 0.833).

#### Self-Perception Profile for Adults

This 50-item self-report questionnaire was developed according to the theoretical framework on Harter's concept of Self and depicts the way that adults perceive themselves in 11 fundamental life areas, as well as adult's global self-worth (41). It is answered on a 4-point scale; higher scores reflect higher perceived competence/adequacy. The Self-Perception Profile for Adults has been translated and validated in Greek by Roussi-Vergou and Zafiropoulou of the Laboratory of Evolutionary Psychology and Psychopathology, University of Thessaly, Greece. All twelve specific domains showed good-to-very good internal consistency in the present study: Sociability (Cronbach's α = 0.780), Job Competence (Cronbach's α = 0.763), Nurturance (Cronbach's α = 0.773), Athletic Abilities (Cronbach's α = 0.769), Physical Appearance (Cronbach's α = 0.838), Adequate Provider (Cronbach's α = 0.736), Morality (Cronbach's α = 0.707), Household Management (Cronbach's α = 0.869), Intimate Relationships (Cronbach's α = 0.818), Intelligence (Cronbach's α = 0.798), Sense of Humor (Cronbach's α = 0.777), Global Self-Worth (Cronbach's α = 0.864).

#### Demographic and Personal Information

Demographic information of participants (age, level of education, nationality, marital status, childhood maltreatment, and partner maltreatment) were obtained though semi-structured interview. The researchers have conceptualized the term of childhood maltreatment as any behavior which includes physical, sexual and verbal abuse and partner maltreatment as any harmful behavior (physical, sexual, verbal, emotional) which occurs in the context of an intimate relationship.

### Statistical Analysis

Associations between quantitative variables were assessed by computing the Pearson product-moment correlation coefficient. The significance of differences was examined using Chi-square test for qualitative variables and independent Student's *t*-test for quantitative variables. All psychometric questionnaires' scores were handled as quantitative variables. Two (2) multiple linear regression analyses were performed in order to adjust for possible confounders the comparison of aggression (dependent variable) (a) between female inmates and non-delinquents and (b) between female inmates who were convicted for violent or non-violent crimes. The following two-step procedure was employed: initially, univariate linear regression models were fitted for each covariate and any variable whose univariable test had a *p* < 0.20 was identified. Next, the identified variables were entered in a multivariable linear regression model and their significance with regard to aggression was assessed with backward stepwise selection procedure (removal *p* >0.05). Both final multiple linear regression models included the statistically significant explanatory variables, as well as the variable representing the participants' group which was retained as it is of substantive importance in this study. The statistical significance level was set at *p* < 0.05 and all statistical analyses were performed using Stata (version 13.0, Stata Corporation, TX, USA).

## Results

Among the 157 female inmates who were included in this study, 60 women were convicted for violent crimes, such as homicides [56 (35.67%)] and serious physical harm [4 (2.55%)], while 97 women were convicted for non-violent crimes, as thefts [18 (11.46%)], white collar crimes [27 (17.20%)], crimes related to drugs [36 (22.93%)], and other [16 (10.19%)] non-violent crimes. The characteristics of the total sample are presented in Table 1.

**Table 1:** Demographic and social characteristics of the 150 non-delinquent women and the 157 female inmates.

**Socio-demographic variables**	**Non-delinquent women (*N* = 150)**			**Female inmates**		
		**Total inmates**	***p*-value**	**Violent crimes (*N* = 60)**	**Non-violent crimes (*N* = 97)**	***p*-value**
**Age [years; M (SD)]**	36.07 (10.09)	35.47 (10.73)	0.617	39.40 (11.22)	33.04 (9.71)	<0.001^*^
**Education [*****N*** **(%)]**			<0.001^*^			0.036^*^
Primary Education	7 (4.67)	42 (26.75)		23 (38.33)	19 (19.59)	
Secondary Education	57 (38.00)	74 (47.13)		24 (40.00)	50 (51.55)	
Tertiary Education	86 (57.33)	41 (26.11)		13 (21.67)	28 (28.87)	
**Nationality [*****N*** **(%)]**			0.002^*^			0.021^*^
Greek	133 (88.67)	118 (75.16)		39 (65.00)	79 (81.44)	
Foreign	17 (11.33)	39 (24.84)		21 (35.00)	18 (18.56)	
**Marital Status [*****N*** **(%)]**			0.016^*^			0.059
Not married/cohabiting	61 (40.67)	56 (35.67)		15 (25.00)	41 (42.27)	
Married	68 (45.33)	60 (38.22)		24 (40.00)	36 (37.11)	
Divorced/separated	17 (11.33)	23 (14.65)		10 (16.67)	13 (13.40)	
Widowed	4 (2.67)	18 (11.46)		11 (18.33)	7 (7.22)	
**Number of children [M (SD)]**	0.98 (1.07)	1.20 (1.40)	0.129	1.28 (1.24)	1.14 (1.50)	0.548
**Childhood maltreatment [yes;** ***N*** **(%)]**	8 (5.33)	29 (18.47)	<0.001^*^	13 (21.67)	16 (16.49)	0.417
**Partner maltreatment [yes;** ***N*** **(%)]**	4 (2.67)	23 (14.65)	<0.001^*^	17 (28.33)	6 (6.19)	<0.001^*^

*Data are presented as Frequency (Column percentage) or Mean (Standard Deviation): M (SD) The significance of differences for the groups (a. non-delinquents vs. total inmates and b. women convicted for violent vs. non-violent crimes) was examined using Chi-square test for qualitative variables and independent Student's t-test for quantitative variables*.

* *Statistically significant*.

*Bold values represent the statistically significant values*.

The relationship of aggression with self-esteem, narcissistic personality traits, and self-perception's factors was examined by Pearson's *r* correlation for non-delinquents, total female inmates, and inmates convicted for violent or non-violent crimes (Table 2). Aggression correlated negatively with self-esteem and positively with narcissistic personality traits within the non-delinquents' and total inmates' groups. Within the group of total inmates, aggression negatively correlated with job competence (*r* = −0.377, *p* < 0.001), nurturance (*r* = −0.175, *p* = 0.028), morality (*r* = −0.280, *p* < 0.001), household management (*r* = −0.245, *p* = 0.002), and intelligence (*r* = −0.191, *p* = 0.016). In non-delinquent women, aggression negatively correlated with nurturance (*r* = −0.308, *p* < 0.001), physical appearance (*r* = −0.228, *p* = 0.005), adequate provider (*r* = −0.265, *p* = 0.001), intimate relationships (*r* = −0.177, *p* = 0.030), and global self-worth (*r* = −0.234, *p* = 0.004).

**Table 2:** Correlations of aggression with self-esteem, narcissistic personality traits and self-perception for non-delinquent women, total female inmates, and for inmates convicted for violent or non-violent crimes.

	**Aggression**							
	**Non-delinquent women (*****N*** **=** **150)**		**Female inmates (*****N*** **=** **157)**					
			**Total inmates (*****N*** **=** **157)**		**Violent crimes (*****N*** **=** **60)**		**Non-violent crimes (*****N*** **=** **97)**	
	***r***	***p*-value**	***r***	***p*-value**	***r***	***p*-value**	***r***	***p*-value**
**Self-esteem**	−0.307	<0.001^*^	−0.277	<0.001^*^	−0.033	0.804	−0.426	<0.001^*^
**Narcissistic personality traits**	0.166	0.043^*^	0.193	0.016^*^	0.289	0.025^*^	0.025	0.184
**Self-Perception**								
Sociability	−0.138	0.092	0.011	0.889	0.170	0.194	−0.069	0.505
Job competence	−0.143	0.081	−0.377	<0.001^*^	−0.276	0.033^*^	−0.424	<0.001^*^
Nurturance	−0.308	<0.001^*^	−0.175	0.028^*^	−0.280	0.031^*^	−0.109	0.289
Athletic abilities	−0.071	0.391	−0.148	0.064	−0.099	0.453	−0.171	0.095
Physical appearance	−0.228	0.005^*^	0.061	0.451	0.152	0.248	0.009	0.933
Adequate provider	−0.265	0.001^*^	−0.035	0.667	−0.039	0.770	−0.009	0.929
Morality	−0.135	0.100	−0.280	<0.001^*^	−0.289	0.025^*^	−0.257	0.011^*^
Household management	−0.120	0.143	−0.245	0.002^*^	−0.387	0.002^*^	−0.180	0.079
Intimate relationships	−0.177	0.030^*^	−0.065	0.419	0.095	0.472	−0.148	0.147
Intelligence	−0.120	0.144	−0.191	0.016^*^	−0.110	0.405	−0.216	0.034^*^
Sense of humor	−0.085	0.302	0.064	0.423	0.114	0.388	0.034	0.745
Global self-worth	−0.234	0.004^*^	−0.127	0.114	0.005	0.973	−0.198	0.052

* *Statistically significant. Correlations by Pearson's r correlation coefficient*.

*Bold values represent the statistically significant values*.

In order to investigate possible differences in aggression, self-esteem, narcissistic personality traits, and self-perception between female inmates and non-delinquents, independent *t*-tests were computed. As shown in Table 3, inmates presented higher aggression (*p* < 0.001) and lower self-esteem (*p* < 0.001) compared to non-delinquents. No statistically significant differences were found regarding narcissistic personality traits *(p* = 0.488). Furthermore, it appeared that the mean (SD) scores of almost all self-perception's subscales were lower in female inmates compared to non-delinquents in a statistically significant manner.

**Table 3:** Comparison of aggression, self-esteem, narcissistic personality traits and Self-perception in non-delinquent women and female inmates.

	**Non-delinquent women (*N* = 150)**	**Female Inmates (*N* = 157)**	**95% Confidence Interval**	***p*-value**
**Aggression**	68.82 (14.95)	78.40 (23.60)	−14.04, −5.12	<0.001^*^
**Self-esteem**	21.65 (4.90)	18.06 (6.19)	2.33, 4.85	<0.001^*^
**Narcissistic personality traits**	10.85 (6.41)	11.43 (8.10)	−2.22, 1.07	0.488
**Self-Perception**				
Sociability	3.15 (0.65)	2.80 (0.69)	0.20, 0.51	<0.001^*^
Job competence	3.17 (0.56)	2.76 (0.68)	0.27, 0.55	<0.001^*^
Nurturance	3.28 (0.56)	3.02 (0.66)	0.12, 0.40	<0.001^*^
Athletic abilities	2.37 (0.68)	2.23 (0.62)	−0.004, 0.29	0.065
Physical appearance	2.81 (0.71)	2.77 (0.70)	−0.12, 0.20	0.626
Adequate provider	2.99 (0.52)	2.55 (0.67)	0.31, 0.58	<0.001^*^
Morality	3.30 (0.50)	2.84 (0.64)	0.33, 0.59	<0.001^*^
Household management	2.94 (0.75)	2.87 (0.72)	−0.09, 0.24	0.380
Intimate relationships	3.09 (0.60)	2.71 (0.72)	0.23, 0.52	<0.001^*^
Intelligence	3.11 (0.53)	2.80 (0.73)	0.16, 0.45	<0.001^*^
Sense of humor	3.18 (0.59)	2.67 (0.71)	0.36, 0.65	<0.001^*^
Global self-worth	3.02 (0.62)	2.60 (0.76)	0.26, 0.58	<0.001^*^

*Values are presented as Mean (Standard Deviation): M (SD)*.

*Independent t-tests in order to determine possible univariate (unadjusted) differences between the mean scores of non-delinquent women and female inmates*.

* *Statistically significant*.

*Bold values represent the statistically significant values*.

With a view to further exploring the aforementioned significant univariate (unadjusted) difference on aggression (dependent variable) between female inmates and non-delinquents we controlled for socio-demographic and psychometric parameters by using backward stepwise multiple regression analysis (Table 4). It emerged that after adjusting for covariates, the aforementioned univariable association lost statistical significance. Furthermore, aggression was affected negatively with age and education (primary education vs. tertiary), negatively with self-esteem and positively with narcissistic personality traits. Aggression was also independently associated with self-perception traits (positively with sociability, but negatively with nurturance, job competence, and athletic abilities).

**Table 4:** Multiple linear regression analysis predicting aggression among 307 female inmates and non-delinquent women.

**Predictors**	**Coefficient (β)**	**95% confidence interval**	***p*-value**
**Group**			
Female inmates (vs. Non-delinquent women)	1.14	−3.31, 5.60	0.614
**Age (years)**	−0.22	−0.41, −0.02	0.029^*^
**Education**			
Secondary (vs. Primary)	−3.35	−9.39, 2.69	0.275
Tertiary (vs. Primary)	−9.13	−15.41, −2.84	0.005^*^
**Self-esteem**	−1.00	−1.43, −0.57	**<** 0.001^*^
**Narcissistic personality traits**	0.61	0.31, 0.91	**<** 0.001^*^
**Self-perception: Sociability**	6.09	2.20, 9.98	0.002^*^
**Self-perception: Job Competence**	−4.39	−8.59, −0.19	0.041^*^
**Self-perception: Nurturance**	−6.59	−10.42, −2.77	0.001^*^
**Self-perception: Athletic Abilities**	−3.41	−6.77, −0.06	0.046^*^
Constant	122.61	106.08, 139.15	<0.001^*^

* *Statistically significant, F_(10, 296)_ = 12.73, p < 0.001*.

Backward stepwise multiple regression analysis (removal p > 0.05), with a view to adjusting the comparison of female inmates and non-delinquent women as regards aggression (dependent variable) for socio-demographic and psychometric parameters. Variables which were initially entered along with women's group were: nationality, education, childhood maltreatment, age, Self-esteem, Narcissistic personality traits, self-perception's subscales: sociability, job competence, nurturance, athletic abilities, adequate provider, morality, household management, intimate relationships, intelligence, and global self-worth.

*Bold values represent the statistically significant values*.

### Comparison Between Violent and Non-violent Inmates

In order to explore possible differences in aggression, self-esteem, narcissistic personality traits, and self-perception between inmates convicted for violent or non-violent crimes, independent *t*-tests were performed. As shown in Table 5, no differences were found in aggression, self-esteem, or narcissistic personality traits. Inmates convicted for violent crimes presented statistically significantly higher mean (SD) scores compared to inmates convicted for non-violent crimes in the following self-perception's subscales: job competence, adequate provider, morality, intelligence, and global self-worth.

**Table 5:** Comparison of aggression, self-esteem, narcissistic personality traits and self-perception among women who were convicted for violent or non-violent crimes.

	**Violent crimes (*N* = 60)**	**Non-violent crimes (*N* = 97)**	**95% confidence interval**	***p*-Value**
**Aggression**	76.00 (22.94)	79.89 (23.99)	−11.54, 3.77	0.318
**Self-esteem**	19.27 (6.56)	17.31 (5.85)	−0.03, 3.95	0.054
**Narcissistic personality traits**	11.85 (8.84)	11.18 (7.64)	−1.96, 3.31	0.613
**Self-perception**				
Sociability	2.85 (0.67)	2.76 (0.71)	−0.13, 0.32	0.412
Job competence	2.92 (0.66)	2.66 (0.67)	0.04, 0.47	0.022^*^
Nurturance	3.10 (0.63)	2.97 (0.68)	−0.09, 0.34	0.265
Athletic abilities	2.30 (0.65)	2.19 (0.60)	−0.10, 0.30	0.317
Physical appearance	2.85 (0.78)	2.72 (0.63)	−0.10, 0.35	0.268
Adequate provider	2.72 (0.61)	2.45 (0.68)	0.06, 0.48	0.014^*^
Morality	3.08 (0.65)	2.70 (0.59)	0.18, 0.58	<0.001^*^
Household management	2.82 (0.67)	2.90 (0.75)	−0.31, 0.16	0.509
Intimate relationships	2.77 (0.70)	2.68 (0.73)	−0.14, 0.32	0.445
Intelligence	3.01 (0.67)	2.68 (0.73)	0.10, 0.56	0.005^*^
Sense of humor	2.67 (0.75)	2.68 (0.69)	−0.24, 0.23	0.970
Global self-worth	2.85 (0.85)	2.45 (0.66)	0.16, 0.65	0.001^*^

*Values are presented as Mean (Standard Deviation): M (SD)*.

*Independent t-tests in order to determine possible univariate (unadjusted) differences between the mean scores of women convicted for violent vs. non-violent crimes*.

* *Statistically significant*.

*Bold values represent the statistically significant values*.

A backward stepwise multiple regression analysis was performed with a view to adjusting the comparison of female inmates who were convicted for violent or non-violent crimes as regards aggression (dependent variable) for socio-demographic and psychometric parameters (Table 6). It was found that lower job competence, higher narcissistic personality traits and a history of childhood maltreatment were independently associated with higher aggression. Furthermore, the effect of the type of offense (violent or non-violent crime) on aggression after adjusting for covariates remained statistically non-significant, as in the preceded univariable analysis.

**Table 6:** Multiple linear regression analysis predicting aggression among 157 women who were convicted for violent or non-violent crimes.

**Predictors**	**Coefficient (β)**	**95% confidence interval**	***p*-value**
**Group**			
Violent crimes (vs. Non-violent crimes)	−1.55	−8.53, 5.44	0.663
**Self–perception: Job Competence**	−13.23	−18.26, −8.19	<0.001^*^
**Narcissistic personality traits**	0.67	0.25, 1.08	0.002^*^
**Childhood maltreatment**			
Yes (vs. No)	11.07	2.44, 19.70	0.012^*^
Constant	105.82	91.05, 120.59	<0.001^*^

* *Statistically significant, F_(4, 152)_ = 10.78, p < 0.001*.

*Backward stepwise multiple regression analysis (removal p > 0.05), with a view to adjusting the comparison of female inmates who were convicted for violent or non-violent crimes as regards aggression (dependent variable) for socio-demographic and psychometric parameters. Variables which were initially entered along with women's group were: nationality, education, childhood maltreatment, age, self-esteem, narcissistic personality traits, self-perception's subscales: job competence, nurturance, athletic abilities, morality, household management, intelligence, and global self-worth*.

*Bold values represent the statistically significant values*.

## Discussion

The present study examines the effect of self-esteem, self-perception, narcissism, and socio-demographic factors on aggression among female inmates and women without criminal record. Our findings indicate that within both groups of women (inmates and non-delinquents), aggression correlated negatively with self-esteem and self-perception and positively with narcissistic personality traits. When inmates were compared to non-delinquents, it emerged that higher aggression as measured by the instruments described could be predicted by higher levels of narcissistic personality traits and sociability, as well as lower age, lower education, lower self-esteem, and lower levels of self-perception items (nurturance, job competence, and athletic abilities). Aggression was not predicted by the participants' group (inmates vs. non-delinquents). Within female inmates, independently of the type of their offense (convicted for violent vs. non-violent crimes), it was found that only lower job competence, higher narcissistic personality traits and a history of childhood maltreatment could predict higher aggression.

The negative relationship of self-esteem and self-perception with aggression which emerged in this study is in accordance with other studies that support the argument that lower self-esteem is connected with higher expression of aggressive behavior (16, 17, 24–26, 36, 42–47). However, other studies have reported an association between high self-esteem and aggression (48, 49), provided mixed results (20) or even indicated absence of any significant association (50).

The positive association of narcissism with aggression gives support to previous research findings which suggest that individuals with inflated ego, indicative of narcissism, are more likely to engage in violent behaviors (29, 49, 51, 52). This research is one of the few that focuses on female inmates. Furthermore, when exploring differences between the two groups with respect to aggression, female inmates presented higher aggression compared to non-delinquents, as indicated with previous research findings (53, 54). This association between criminal record and aggression after adjusting for socio-demographic and psychometric parameters was not statistically significant. Especially, our results indicate that it is lower self-esteem, lower self-perception and higher narcissism along with lower age and education that independently predict higher aggression in women for both groups. Female criminality by itself in our study is not linked to aggression as by Buss & Perry Aggression Questionnaire (BPAQ).

The aforementioned findings point out the important role of self-esteem and self-perception in aggression. Indeed, Donnellan et al. in a sample of children and adolescent teenagers of both sexes found a strong association between low self-esteem and aggression, delinquency and anti-social behavior in children and teenagers (24), while Dmitrieva et al. found that low self-esteem is associated with gang membership during adolescence in gang members adolescent mainly male and female (44). In a study of female inmates, low self-esteem was related to higher levels of aggression (36). Another study investigating self-esteem of inmates of both sexes in two medium-security prisons supported the relationship between crime and low self-esteem (45). It has been suggested that aggression may provide individuals with low self-esteem with an increased sense of power and independence, or serve as an attention seeking behavior which enhances self-esteem. Possibly, individuals with low self-esteem externalize blame for their problems and failure to protect themselves against feelings of inadequacy, inferiority, and shame, which leads to aggression toward others (26). According to Rogers, failure to develop positive self-esteem leads to psychological problems and induces aggressive behavior (46). Horney asserted that an inferiority complex and a sense of humiliation (including low self-esteem) may increase aggressiveness and lead toward antisocial behavior (47).

Although self-perception in female inmates has not been widely investigated, there is evidence that it is associated with female criminality (16). According to McClellan et al. victimization of women in young age leads to depression, and substantially affects their self-perception and the course of criminal activity during adulthood (55). Bloom and Owen support that female criminality is a product of self-perceptions constructed during childhood (17).

The positive association of narcissism with aggression gives support to previous research findings which suggest that individuals with inflated ego, indicative of narcissism, are more likely to engage in violent behaviors (49, 52). Vaughn et al. found that narcissism is related to an increased number of arrests and armed assaults, whereas Johnson et al. suggested that the presence of narcissistic symptoms early in adolescence is a predictive factor for the manifestation of violent criminal behavior in the beginning of adulthood (51, 56). This finding was furthered supported by Barry et al. who found that teenagers with criminal record tend to be more narcissistic compared to general population (49). Hepper et al. also found that narcissism as a personality trait leads to a greater likelihood of involvement in criminal behavior (29).

Moreover, it emerged that younger age was independently associated with higher aggression, a finding concordant with international scientific literature (57). Although there is no sufficient research evidence regarding the impact of age on aggression among female inmates, it may be suggested that young age contributes to the expression of aggressive behaviors either due to increased self-esteem or beliefs of omnipotence, which have not yet been counterbalanced by life experiences. In fact, women who commit homicide are mainly young, aged 31 on average. Impulse control decision making and maturity are rather achieved later in the course of life (58, 59).

It is noteworthy that among women, education is inversely related to aggression and thus it is a protective factor against aggression. Therefore, it could be suggested that promotion of education may serve as a preventive strategy against aggressive behavior. Studies support that a higher education contributes and enhances the process of socialization, the development of personality, as well as the professional development and therefore reduces the risk for delinquent behavior (60). Schooling significantly reduces the probability of incarceration. Differences in educational attainment between black and white men explain 23% of the black white gap in male incarceration rates. Furthermore, findings on incarceration using FBI data show that the biggest impacts of education are associated with murder, assault, and motor vehicle theft (61).

These results could be interpreted by the theory of psychodynamic mask model (33, 34) according to which narcissism appears to act as a mask for their low self-esteem, which is related to higher rates of aggression. Myers and Zeigler-Hill found support for the psychodynamic mask model of narcissism by utilizing the bogus pipeline technique to reveal how narcissistic individuals have lower self-esteem than they overtly express (35). Furthermore, Barnett and Powell found in their research that Self-esteem mediates narcissism and aggression among women (62). Donnellan et al. found that the relationship between low self-esteem and aggression is independent from narcissism (24).

Concerning the comparison among female inmates convicted for violent or non-violent crimes, no differences were found in aggression, self-esteem or narcissistic personality traits. Inmates convicted for violent crimes compared to inmates convicted for non-violent crimes presented higher scores in certain self-perception dimensions such as job competence, adequate provider, morality, intelligence, and global self-worth. These findings add to the scarce literature on female criminal aggression and support the notion that female might differ substantially from male aggressive criminality.

Statistics have been consistently showing that men commit more criminal acts than women (63). Self-reported delinquent acts are also higher for men than women (64). Burton et al. found that low levels of self-control are associated with criminal activity (65). Females usually commit homicidal acts in the context of an intimate relationship or against individuals with whom they are emotionally involved driven by personal gain (66, 67). In contrast to men, women rarely commit violent aggressive behaviors toward strangers (68).

After adjusting the comparison of female inmates convicted for violent or non-violent crimes as regards aggression for socio-demographic and psychometric parameters, it was found that lower job competence, higher narcissistic personality traits and a history of childhood maltreatment were independently associated with higher aggression. Furthermore, aggression remained no significantly different between inmates convicted for violent crimes or non-violent crimes.

It is noteworthy that among inmates, given the aforementioned factors, self-esteem does not independently predict aggression, as it did among the total women sample. Therefore, it seems that although for the total sample of women self-esteem affects significantly the course of aggression, this is not the case for female offenders, where narcissism and childhood maltreatment seem to have a leading role in the manifestation of aggression.

Concerning the fact that the type of crime (violent or non-violent) had no effect on aggression due to the paucity of relevant studies in females, this finding is not comparable to previous research. Only one study compared aggressive behavior in prison male inmates and non-prison inmates in Nigeria using another questionnaire showed significant differences in physical aggression between the two groups with the group of prisoners scoring higher on aggression than non-prisoners (69). One possible explanation for the absence of difference with respect to aggression between violent and non-violent female offenders is that most of the women convicted for violent crimes, committed homicide in the context of inter- partner violence. Research suggests that female criminality is reactive, and that aggressive behaviors are endorsed for survival purposes and as a coping mechanism rather than biologically driven (54). Furthermore, women are more likely than men to experience acts of aggression as expressive (a loss of self-control) than as instrumental (control over others). This might possibly arise from gender differences in behavioral restraint. It is suggested that women have better inhibitory control and therefore aggressive behavior occur less frequently and experienced as more emotionally. Women can tolerate higher levels of anger before inhibitory control is infringed (70). Our findings lend weight to the notion that violence in women might present through different pathways than men.

Another possible explanation for the absence of difference in aggression among female inmates could be related with issues that pertain to the conceptual framework of aggression and its measurement. It is accepted that there are other types of aggression such (indirect) prominent among women, recently attracting research attention (6, 71).

Research has indicated that both men and women under provoking conditions could be equally violent (5, 72, 73). Men tend to perceive environmental stimuli as more provoking compared to women (74).

The present study found that inmates, victims of abuse during childhood tend to have higher levels of aggression independently of the type of offense, narcissistic traits, and job competence. It is common ground among relative scientific studies that female criminality is strongly associated with women victimization (54). Literature suggests that aggressive women have been witnesses or victims of domestic violence and abuse either during childhood or adulthood (13, 14, 75–77). Similarly, the victimization of women increases significantly the likelihood of their involvement in criminal activity (55, 78–81). In fact almost, half of the female prisoners have been victims of abuse with 75% of them having suffered physical abuse and 65% sexual abuse (55, 81–89).

Additionally, the strong impact of narcissism on aggression can also be conceptualized within the context of victimization, since there is a reported positive relationship between trauma experiences and the development of fragile self-image (90). According to Chesney-Lind victimization especially during childhood and by family members or trusted individuals increases the risk of involvement in criminal activity in adulthood, the likelihood of suffering depression and low self-esteem, which all lead to a vicious circle triggering further victimization and criminality (80). Covington argues that theories of female criminality should take into consideration the significant impact of past trauma, victimization, substance abuse, mental health issues, and significant relationships (78). Our study strengthens this approach.

Our findings should be considered with regard to study limitations. The present study is cross-sectional and therefore does not account for any changes due to time or shifting in individual differences. The use of self-report questionnaires comes along with notable issues, among others the need for social acceptability and reporting biases. The sample of the general population (absence of criminal record) was pooled from one region—prefecture of Attica, which could impact the generalizability of results. Furthermore, due to the fact that certain criteria should be met for the individuals in order to be able to participate in the present study (language efficiency, informed consent) there could be a selection bias due to the non-random sampling. Finally, in order to achieve high external validity (by employing actual individuals with criminal record) the relatively modest sample size may have limited the statistical power to detect modest but meaningful associations as statistically significant.

## Conclusion

The present study provides evidence on the important role of self-esteem, self-perception, and narcissism, as well as certain socio-demographic parameters on aggression among women. As evident, for all participants independently of the manifestation of criminal behavior, lower self-esteem, lower self-perception (certain parameters), higher narcissistic traits, lower age, and education could predict aggression. Within female inmates, independently of the type of their offense, lower job competence, higher narcissistic personality traits and a history of childhood maltreatment could predict higher aggression. It is the presence of narcissistic traits which predict aggression rather than criminality in general, including violent and non-violent crimes.

This to our knowledge is the first study comparing alone female inmates with a general population sample on the aforementioned parameters and lends support to the notion that female aggression is different from male and findings from male samples might not apply to females.

Further studies should be conducted in order to assess the directionality and causality among those variables. Hopefully our results will contribute to a better understanding of the female aggression and the development of gender—sensitive therapeutic interventions with a specific concentration toward self-esteem empowerment, tailor made services for abused and battered women and therapeutic programs for personality disorders.

## Author Contributions

GK was a co-designer of the study, participated in data collection and processing and wrote the manuscript. IM was a co-designer and drafted the manuscript. VE did the statistical analysis and interpretation of the data. FK participated in data collection and processing. DT participated in revision of the manuscript. RG revised the manuscript. AD was a co-designer of the study, drafted, and made critical and substantial corrections in the manuscript. All authors read and approved the final manuscript.

### Conflict of Interest Statement

The authors declare that the research was conducted in the absence of any commercial or financial relationships that could be construed as a potential conflict of interest.
